# Behavioural-variant frontotemporal dementia: an update

**DOI:** 10.1590/S1980-57642013DN70100003

**Published:** 2013

**Authors:** Olivier Piguet, John R. Hodges

**Affiliations:** 1 Neuroscience Research Australia, Barker St, Randwick NSW 2031, Australia. School of Medical Sciences, the University of New South Wales, Sydney, Australia. ARC Centre of Excellence in Cognition and its Disorders, the University of New South Wales, Sydney, Australia.

**Keywords:** frontotemporal dementia, genetics, cognition, social cognition, neuroimaging

## Abstract

Behavioural-variant frontotemporal dementia (bvFTD) is characterised by insidious changes in personality and interpersonal conduct that reflect progressive disintegration of the neural circuits involved in social cognition, emotion regulation, motivation and decision making. The underlying pathology is heterogeneous and classified according to the presence of intraneuronal inclusions of tau, TDP-43 or occasionally FUS. Biomarkers to detect these histopathological changes in life are increasingly important with the development of disease-modifying drugs. Gene mutations have been found which collectively account for around 10-20% of cases including a novel hexanucleotide repeat on chromosome 9 (*C9orf72*). The recently reviewed International Consensus Criteria for bvFTD propose three levels of diagnostic certainly: possible, probable and definite. Detailed history taking from family members to elicit behavioural features underpins the diagnostic process with support from neuropsychological testing designed to detect impairment in decision-making, emotion processing and social cognition. Brain imaging is important for increasing the level of diagnosis certainty. Carer education and support remain of paramount importance.

## Abstract (pt)

Demência frontotemporal-variante comportamental (DFTvc) é caracterizada por mudanças insidiosas de personalidade e conduta interpessoal, que refletem a desintegração progressiva de circuitos neurais envolvidos em cognição social, regulação emocional, motivação e tomada de decisão. O substrato patológico é heterogêneo e classificado de acordo com a presença de inclusões intraneuronais de proteína tau, TDP-43 ou, ocasionalmente, de FUS. Biomarcadores capazes de detectar estas alterações histopatológicas durante a vida vêm ganhando importância com o desenvolvimento de drogas específicas modificadoras da doença. Algumas mutações genéticas já foram encontradas, sendo em conjunto responsáveis por 10-20% dos casos, incluindo a recentemente descrita repetição de hexanucleotídeo no cromossomo 9 (*C9orf72*). A versão revisada dos Critérios Internacionais do Consenso em DFTvc propõe três níveis de certeza diagnóstica: possível, provável e definida. História clínica detalhada obtida com familiares, para identificar as alterações de comportamento características, auxilia no diagnóstico, juntamente com o apoio de avaliação neuropsicológica dirigida à detecção de comprometimento em tarefas de tomada de decisão, processamento emocional e cognição social. A neuroimagem é importante para aumentar o grau de certeza diagnóstica. Educação e suporte dos cuidadores continuam sendo medidas de extrema relevância.

## INTRODUCTION

Frontotemporal dementia (FTD) is the clinical diagnostic label now preferred to describe patients with a range of progressive dementia syndromes associated with focal atrophy of the orbitomesial frontal and anterior temporal lobes. Epidemiological studies suggest that FTD is the second most common cause of young onset dementia after Alzheimer's disease (AD).^1,2^ Two independent studies from the UK revealed a prevalence of around 15 per cases per 100,000 population aged 45 to 64 with broad confidence intervals (8 to 27).^1^ Although regarded as a rare cause of dementia after 65 years, FTD may be more common than assumed because older adults rarely undergo the types of investigation needed to establish a confident *in vivo* diagnosis and are not followed to autopsy.

Unlike AD, both the clinical profile and underlying pathology are heterogeneous in FTD. Two broad presentations are recognised: progressive deterioration in social function and personality, known as bvFTD (or sometimes simply FTD) and insidious decline in language skills, known as primary progressive aphasia which can, in turn, be subdivided according the predominant pattern of language breakdown into progressive nonfluent aphasia and semantic dementia.^3-5^ The syndrome of FTD overlaps with motor neurone disease (MND) both clinically and pathologically, and with a number of the extrapyramidal motor disorders. Around 10% of patients with FTD develop clinical and neurophysiological evidence of MND.^6,7^ and likewise patients with MND show behavioural and/or language changes which, in some instances, are severe enough to qualify for a diagnosis of FTD.^8^ Of the extrapyramidal disorders, corticobasal degeneration and progressive supranuclear palsy show substantial overlap with FTD and share the finding of abnormal tau pathology.^9^

This review cannot cover every facet of this rapidly evolving field and focuses on the clinical aspects of bvFTD within the context of recent pathological and genetic discoveries. Readers are referred to recent authoritative reviews on the language presentations of FTD.^3,4^

## PATHOLOGY

The subtypes of underlying pathology in patients with FTD are classified on the basis of the pattern of protein accumulation and are referred to collectively as frontotemporal lobar degeneration (FTLD).^10^ At *postmortem*, cases share, by definition, the finding of bilateral frontotemporal atrophy with neuronal loss, microvacuolation and a variable degree of astrocytic gliosis. The progression of this atrophy has been examined by mapping the pattern in patients with different disease duration.^11^ Initially, mesial and orbital frontal regions bear the brunt of the atrophy followed by the temporal pole, hippocampal formation, dorsolateral frontal cortex and the basal ganglia. This pattern of progression of atrophy has been shown to relate to the volume of cortical and subcortical regions^12^ and underlying neuron loss.^13^

Inclusions of the microtubular binding protein tau are present in approximately 40% of cases (FTLD-tau).^14^ Tau positive cases include the subset with mutations of the microtubule associated phosphoprotein tau (*MAPT*) gene. Further sub-classification is based on morphologic criteria and the predominance of either tau with three microtubule binding repeats (3R tau) or four microtubule binding repeat (4R tau).^10^ The majority of the remaining cases are tau negative ubiquitin positive and have inclusions comprising the TAR DNA binding protein of 43-kDA or TDP-43 (FTLD-TDP). A minority (around 5 to 10%), which are both tau and TDP-43 negative, harbours inclusions of FUS, fused in sarcoma protein (FTLD-FUS).^14,15^ A small proportion of cases has either no inclusions (FTLD-ni) or shows ubiquitin inclusions which TDP-43 and FUS negative (FTLD-UPS)^14^ suggesting that additional protein abnormalities will be found in FTLD.

In bvFTD, any of the histological variants can be found, with an approximately 50-50 split between FTLD-tau and FTLD-TDP,^16,17^ and a small proportion of FTLD-FUS cases.^18^ With the advent of potential diseasemodifying therapies, ascertainment of a pathological diagnosis *in vivo* will be increasing important. As yet, no reliable method of determining pathology in life exists.

## GENETICS

Up to 40% of patients with FTD are said to have a family history of dementia^2^ but the high community prevalence of non-FTD dementia means that many of the elderly family members included in such estimates almost certainly have other causes of dementia. Patients with an autosomal dominant pattern (affected first degree relatives across two generations) account for only 10% of cases.^19^ The strength of family history is highly predictive in that mutations can now be demonstrated in the majority of patients with two or more first degree relatives with a dementia syndrome compatible with FTD.^19^ Mutations of the *MAPT*, and the progranulin (GRN) genes each account for 5-11% of total FTD cases.^19^ Linkage studies of familial FTD-MND clusters indicated a common locus in the region of chromosome 9p13.2– 21.3.20In 2011, the responsible mutation, a novel hexamino acid expansion termed *C9orf72* was identified^21,22^ which appears to be the most common gene abnormality in FTD.^23^ It is particularly associated with familial FTD-MND but is also found in patients with bvFTD, some of whom may have a family history of MND.^23^ Patients with this mutation appear to have a particularly high rate of psychotic features which are otherwise rare in FTD.^24,25^ In our own experience, screening 89 patients with FTD syndromes revealed 10% with the C9orf72 mutation^24^ compared to an earlier similar study which found a prevalence of the GRN mutation of around 4%.^26^ The three most common mutations – *MAPT*, GRN and *C9orf72* together account around three-quarters of familial FTD and FTD-MND cases. Mutations of the genes encoding for TDP -43 (TARDBP) and *FUS*, recognised as a cause of familial ALS, have also been identified in a small number of cases of FTD-ALS^27,28^ but seems rare in uncomplicated FTD.^19^ Rare genetic mutations causing FTD include valosin-containing protein (*VCP*) and charged multivesicular body protein 2B (*CHMP2B*). Mutation of the *VCP* gene causes FTD in association with inclusion body myopathy and Paget's disease of bone,^29^ whereas the *CHMP2B* gene mutation is mostly confined to a large Danish cohort with FTD.^30,31^

From a practical perspective, a detailed family history should be taken in all patients with suspected FTD bearing in mind the overlap between MND and FTD, that a diagnosis of FTD or Pick's disease was rarely made in the past and the phenotypic variability within families with gene mutations: one member may present with bvFTD and others have a progressive aphasic syndrome or corticobasal syndrome. Based upon a comprehensive analysis of the frequency gene mutations according to strength of family history and clinical syndrome in a large clinical cohort^19^ and recent findings related to the *C9orf72* gene expansion, we recommend that patients with one or more first -degree relatives with a disease within in the FTD spectrum, including MND, be screened for *MAPT, GRN* and *C9orf72* gene mutations after appropriate counselling in a clinical genetics setting. If the patient has FTD-MND, or a family history of MND, or features of psychosis, then screening for *C9orf72* should be conducted first. Those with an informative family history that reveals no affected relatives can be confidently reassured and need not undergo gene screening. It should be noted that the age of onset in patients with *MAPT* gene mutations is almost always below 65 whereas those with *GRN* mutations are often older.^19^

## BEHAVIOURAL FEATURES OF BVFTD

Insidious changes in personality, interpersonal conduct and emotional modulation characterise bvFTD and reflect progressive disintegration of the neural circuits involved in social cognition, emotion regulation, motivation and decision making.^32-34^ The onset is typically difficult to pinpoint. Since insight is limited, or absent, an interview with a close family member to elicit the nature of the early symptoms and their progression is vital. The assessment and diagnosis has been greatly assisted by the development of carer based questionnaires designed to document the range of symptoms found in the dementia, notably the Neuropsychiatric Inventory,^35^ Cambridge Behavioural Inventory^36^ and the Frontal Behavioural Inventory.37All of the features found in bvFTD can occur in other dementias but it is their predominance and early emergence that typify bvFTD (Table 1).

**Table 1:** Symptoms characteristic of behavioural-variant frontotemporal dementia.

Symptom	Clinical characteristics
Apathy	Very common; manifests as inertia, reduced motivation, lack of interest in previous hobbies, and progressive social isolation
Disinhibition	Often coexists with apathy; produces impulsive actions leading to overspending, tactless or sexually inappropriate remarks, and a range of socially embarrassing behaviour.
Repetitive or stereotypic behaviour	May be apparent with perseveration, and a tendency to repeat phrases, stories or jokes.
Hoarding	When severe can result in squalor.
Mental rigidity	Common; patients may have difficulty adapting to new situations or routines.
Blunting of affect	Frequent reduction in range of emotional expression; Elevation of mood resembling hypomania can also be seen.
Changes in eating behaviour	Impaired satiety; change in preferences towards sweet food; common dysregulation of food intake.
Loss of empathy	Common early symptom; lack of empathy towards others; inappropriate or subdued grief reaction.
Other symptoms	New onset pathological gambling; hyper-religiosity (rare).

Psychotic symptoms such as delusions, paranoid ideation and hallucinations are relatively rare in FTD, except in patients with FTD or FTD-MND associated with the *C9orf72* gene expansion in whom a prevalence of up to 40% of psychosis has been reported.^24,25^ Psychosis has also been reported in young-onset patients with FTLD-FUS^6,38^ who can present with florid behavioural symptoms. In these patients, their age of onset is often exceptionally young with an average of 41 years and a positive family history appears rare in keeping with the absence of FUS gene mutations in this group.^18^

Social disinhibition, euphoria, stereotypical or aberrant motor behaviour, and changes in eating preference are the features that most clearly discriminate bvFTD from AD.^36^.^39^ Increased behavioural changes have been associated with disease severity.^40^ Agitation, disinhibition and irritability also seem to be more frequent in the later stages,^41^ while restlessness and hyperorality are present throughout the disease.^42^

In summary, behavioural assessment is a central component of the examination of patients with potential bvFTD and appears more sensitive in distinguishing bvFTD from AD than standard cognitive testing. Despite a considerable increase of our knowledge of the behavioural changes in bvFTD, which are at the root of so much carer distress, much remains uncertain particularly concerning their specificity, neural basis and their relation to underlying pathology.

## THE BVFTD PHENOCOPY SYNDROME AND IMPLICATIONS FOR DIAGNOSTIC CRITERIA

The diagnosis of bvFTD is by no means an easy task in the early stages and many of the symptoms overlap with those seen in psychiatric disorders as well as other dementias.^38^ It is also increasingly apparent that a subset of patients who present with the clinical features of bvFTD do not progress to frank incapacitating dementia.^43^ Such patients are almost always men and they remain stable over many years or improve.^44,45^ The symptom profile as reported by family members is identical except that activities of daily living are less disrupted.^45,46^ A number of features distinguish these non-progressor or phenocopy cases from those with true FTD, notably normal or marginal impairment on neuropsychological tests of executive function, preserved memory and social cognition, a lack of overt atrophy on MRI, and normal metabolic (PET) imaging brain.^43-45,47^

To qualify for a diagnosis of the phenocopy syndrome we recommend that patients should remain stable without evidence of brain atrophy or decline on cognitive tasks with maintenance of ADLs over a period of three years. Diagnostic caution is advised in patients with a possible bvFTD diagnosis as some of these do progress over time. In our experience, those who eventually fall in the "phenocopy" category remain stable over many years. In Cambridge, some have been observed over a decade without progression to frank dementia.

The aetiology of the phenocopy syndrome is a matter of debate. A proportion of patients appear to have a developmental personality disorder in the Asperger's spectrum with decompensation due to altered life circumstances (personal observation). Some may have a chronic low-grade mood disorder, but others remain a mystery, although a genetic aetiology may be a possibility.^48^ Within the international consensus criteria for bvFTD, phenocopy cases qualify for a diagnosis of possible bvFTD, on the basis of the presence of three core behavioural or cognitive features (social disinhibition, apathy, loss of empathy, stereotypic behaviours or alterations in eating pattern, neuropsychological deficits indicative of frontal executive dysfunction). A diagnosis of probable bvFTD, however, is not applicable, as it requires evidence of functional decline and unequivocal neuroimaging abnormalities.

## NEUROPSYCHOLOGY OF BEHAVIOURAL-VARIANT FTD

**Cognition in bvFTD.** Early in the disease process, bvFTD patients may perform relatively well on formal neuropsychological tests despite the presence of significant personality and behavioural changes.^49^ The Mini- Mental State Examination (MMSE) is insensitive but the Addenbrooke's Cognitive Examination (ACE and ACE-Revised) appears to detect around 90% of cases at presentation.^50^ The prototypical cognitive profile is one of relatively preserved language and visuospatial/constructive abilities. Whether bvFTD patients exhibit executive dysfunctions remains contentious,^51,52^ and has been complicated by the inclusion of phenocopy cases (see above). Such deficits, however, constitute a central diagnostic feature of the newly proposed clinical diagnostic criteria.^52^ The combination of specific tests (e.g., Digit Span backward, the Hayling test of response inhibition and the short version of the executive and social cognition battery) may help differentiate these cases, as tests are typically abnormal in patients with true bvFTD and normal in phenocopy cases.^51,53^

The current international consensus criteria for bvFTD advocate a relative sparing of episodic memory.^54^ A proportion (10-15%) of patients with pathologically confirmed FTLD, however, present with severe amnesia.^16,55^ Deficits in episodic memory appear more common than previously reported,^56^ with deficits being, in some instances, as severe as in AD on tests of episodic memory, even after accounting for disease severity.^57^ These deficits are found not only on tests of anterograde memory but also on tests of autobiographical memory and tests of future thinking.^58,59^

The existing evidence indicates that no specific cognitive profile appears to be associated with bvFTD early in the disease, although careful cognitive evaluation will reveal deficits, generally in the domains of executive function and episodic memory. With disease progression, the pattern of deficits becomes less distinct from other FTD subtypes, notably semantic dementia.^9^

The difficulty in identifying profiles of cognitive deficits specific to bvFTD has led to an interest in aspects of social cognition (notably Theory of Mind), emotion recognition and complex problem solving, as well as the use of naturalistic tasks (i.e., tasks reflecting cognitive and non cognitive demands more akin to daily activities). The orbitomesial frontal cortices are critical for performance in these domains and lesions within these brain regions have been shown to impact negatively on tests measuring these cognitive constructs.^60^ Not surprisingly, bvFTD patients have been found to be impaired on tasks such as the Iowa Gambling task, Go- NoGo, or Reversal learning.^61,62^

**Emotion recognition and social cognition in bvFTD.** A striking impairment in emotion detection and recognition is evident early in the course of bvFTD and appears to be most pronounced for negative emotions, such as fear, sadness, anger and disgust.^63^ Disorders of emotion detection and regulation are part of the clinical diagnostic criteria for the disease (i.e., early emotional blunting; early decline in social interpersonal conduct). It is important to note, however, that such deficits are not limited to this subtype of FTD and are also present in semantic dementia.^64^ Difficulties in detecting and understanding emotions are observed with static (photos) or dynamic (films) visual stimuli,^65,66^ voices,^67^ emotional words^68^ or even music.^69^ Importantly, physiological responses (e.g., skin conductance) to some emotional stimuli appear preserved.^70^ Deficits have also been observed in detecting more complex emotions, such as embarrassment.^71,72^ Some of these deficits are modulated by coexisting cognitive deficits^73,74^ and might be amenable to retraining to enhance their recognition and improve interpersonal relationships.

Patients with bvFTD patients are also impaired on many aspects of social cognition. For example, the often reported feature of lack of empathy and coldness is confirmed on formal testing.^75^ Theory of mind is impaired in bvFTD, as exemplified by defective ability to infer intention and mental states in others, to take someone else's point of view,^62,76,77^ detection of social faux pas,^76^ discrimination of sincere from sarcastic exchanges^47,78^ and understanding of situations requiring moral judgment.^79^ These deficits have been recently described as a failure to process contextual information.^80^ While most of the tasks developed remain in the research arena, well-validated tests of emotion and sarcasm detection exist^81,82^ which will hopefully become part of the standard cognitive evaluation in suspected bvFTD.

## NEUROIMAGING IN BVFTD

In most cases, atrophy of the mesial frontal, orbitofrontal and anterior insula cortices can be visually observed on magnetic resonance imaging (MRI) acquired in the coronal plane.^83-85^ A normal MRI to visual inspection does not, however, completely exclude a diagnosis of bvFTD, as the changes may be subtle in the early stages.

Automated quantitative methods including voxelbased morphometry and cortical thickness mapping have revealed selective atrophy of the anterior cingulate and frontal insula cortices early in the course of bvFTD.^85,86^ The anterior cingulate-frontal insula complex contains the von Economo cells, a unique population of neurons thought to be involved in the development and maintenance of social cognition, which are depleted in patients with bvFTD coming to autopsy.^34,88^ Significant changes in structural and functional connectivity among the regions most sensitive to atrophy in bvFTD compared to healthy controls or patients with other dementia syndromes have also been reported.^87,89^ Patterns of grey matter atrophy may be predictive of the underlying pathological process in bvFTD,^18,90,91^ although patterns of atrophy appear to relate more closely to clinical features than to specific pathologies.^92^

Brain atrophy in bvFTD is also present in subcortical brain regions, including amygdala, hippocampus, caudate, striatum, putamen, thalamus, and hypothalamus, ^93,94^ accompanied by reduction in functional and structural connectivity among a number of subcortical and cortical structures.^89,95^

In contrast to the well-documented cortical grey matter changes, presence and severity of white matter changes in bvFTD have only recently been investigated. Frontal lobe white matter volume reduction largely parallels the atrophy in the adjacent grey matter in bvFTD with different subtypes of FTD showing specific patterns of white matter atrophy.^96-98^ Using diffusion tensor imaging (DTI), which is an index of changes in the microstructure organisation of the white matter, studies have successfully differentiated bvFTD from AD, as well as the different FTD subtypes.^96,98,99^ Patients with bvFTD appear to show a selective reduction in some white matter tracts (superior longitudinal fasciculus, uncinate fasciculus, cingulum tracts and genu and splenium of the corpus callosum), particularly those within the frontal lobe (e.g., genu of the corpus callosum) or those connecting frontal and temporal brain regions (e.g., uncinate fasciculus).^96,99,100^

Functional neuroimaging techniques, such as hexamethyl propyleneamine oxime-single-photon emission computed tomography (HMPAO-SPECT) or (^18^F)-fluorodeoxyglucose positron emission tomography (FDGPET) are increasingly used to help with the diagnosis of bvFTD. Frontal hypoperfusion is present on SPECT in bvFTD and differs from that observed in AD, which is predominant in the temporoparietal and posterior cingulate cortices.^101^ Although SPECT appears to be more sensitive than structural MRI in detecting early pathological changes in bvFTD, quantification and specificity of these changes are not established. Hypometabolism on FDG-PET is detected consistently and reliably in the frontal brain regions in bvFTD patients compared to AD patients who show posterior cingulate hypometabolism early in the disease process.^102^ These changes are detected before any changes are visible on structural MR images making FDG-PET the most sensitive diagnostic tool currently available. It is also particularly useful to help identify phenocopy cases who will show preserved frontal metabolism. In patients showing clear brain atrophy on structural MR images, however, little additional diagnostic benefit is gained by conducting a PET scan, as focal atrophy is a positive predictive marker of FTD.

The novel PET technique, employing the β–amyloid detecting [11C] Pittsburgh Compound B (PIB), shows promising results in discriminating AD and FTD cases, ^103,104^ particularly those presenting with language deficits rather than behavioural changes. Its use as a routine test remains to be established but its clinical applicability is evident as therapeutic interventions are being developed that are likely to be pathology specific.

In summary, neuroimaging investigations in the diagnosis of bvFTD are powerful tools, which can reliably differentiate bvFTD from other FTD subtypes and from other dementia syndromes, and can corroborate clinical diagnostics based on neuropsychiatric symptoms.

## MANAGEMENT

No disease specific treatment interventions for FTD currently exist. Treatment largely remains supportive and involves a combination of non-pharmacological and pharmacological measures, aimed at reducing the effect of distressing symptoms.^105^ The role of pharmacological interventions in FTD remains uncertain, and only small and often conflicting treatment trials have been conducted thus far that have also failed to consider impact on carer stress as a major outcome variable. Selective serotonin reuptake inhibitors (SSRIs) have been used to treat disinhibition and challenging behaviours, but evidence for their use remains contradictory.^106,107^ Atypical antipsychotics such as olanzapine have been used for patients with prominent agitation, aggressive behaviour or psychosis.^108^ Anticholinesterase inhibitors, the mainstay of AD therapy, do not have an established role in the treatment of FTD. One study reported improvement in measures of behavioural disturbance and carer stress with rivastigmine,^109^ however deterioration in neuropsychiatric symptoms without cognitive improvement was demonstrated with donepezil.^110^ Two recently reported double-blind, placebo-controlled, trials of memantine, a non competitive inhibitor of NMDA receptors, failed to show any significant benefit in terms of symptom improvement with a suggestion of cognitive worsening.^111,112^ A number of drugs under development attempt to reduce aggregation of tau or TPD-43 and hence slow the fundamental pathological process in FTD.^105,113^

Limited information is available on the effectiveness of systematic caregiver intervention, although a recent pilot study which employed the antecedent-behaviourconsequence (ABC) model^114^ produced an encouraging reduction in caregiver distress and improved coping strategies.^115^ Studies have confirmed the clinical impression that caregiver burden is much greater in FTD than in AD.^116-118^ Behavioural changes, rather than level of disability, appear to be correlated with caregiver distress and burden in bvFTD,^116^ although a younger age at disease onset and disease severity also appear to impact on burden of care.^119,120^ Evidence indicates that caregiver health is a major contributor to carer stress, with depression accounting for 58% of the variance of stress scores on FTD caregivers.^117^ It seems the key for reducing caregiver stress lies in increasing their understanding of the symptoms and ways of dealing with challenging behaviours.

## CONCLUSIONS AND FUTURE DIRECTIONS

Knowledge of the clinical presentation in bvFTD and its pathological processes has improved dramatically over the past 20 years. Clinicians have become more aware of this disabling neurodegenerative condition affecting individuals who are not uncommonly still in the workforce or with young children. Careful medical history and information from family members, combined with clinical investigations, neuropsychological testing including investigations of social cognition have increased case identification. Sensitivity has also improved with the use of advanced structural and functional neuroimaging techniques. The major challenge that remains, however, is to improve the prediction of the underlying neuropathology in bvFTD patients during life. Efforts to identify potential disease biomarkers for the disease are promising but will require further investigations. This line of research will become particularly relevant as disease-modifying agents are being developed.
